# Reflections from a Patient and Carer on Involvement in Research and Integrating Care in the Health System

**DOI:** 10.5334/ijic.3088

**Published:** 2017-06-27

**Authors:** Frank Hanson, Rhene Hanson

**Affiliations:** 1 University of Toronto, CA

**Keywords:** Patient-engagement, Caregivers, Carers, Primary Care, Canada, Integrated Care

## Introduction

Researchers are increasingly being asked to include patient engagement in their research programs. Some organizations, such as the UK-based Involve [1], have begun to organize approaches to engage patients in research. This article provides a perspective on front-line involvement of a patient and caregiver in a research project focused on integrated care.

My name is Frank Hanson and I have been diagnosed with multiple chronic medical issues. I have been married to my caregiver Rhene for over 50 years and we are both in our seventies. In this article we offer our perspective as patient and caregiver advisors engaged in the multi-year iCOACH (implementing Integrated Care for Older Adults with Complex Health needs) research project. Based on our experience thus far, we have provided some suggestions for including patients and caregivers more effectively. We also share a few of our thoughts and suggestions after several decades of navigating our way through the Ontario health care system.

## Participating as a Patient and Caregiver in a Research Project

We were first engaged as participants in a small research study that included interviews with patients aged 65 and over living with multiple chronic conditions, as well as their caregivers and physicians, to ask about goals of care [2,3,4]. We were subsequently asked to participate in future research, and we agreed. After a meeting with the Principal Investigator to discuss what was being asked of us, we were pleased to participate as patient and caregiver advisors.

We have been recipients of many benefits of the health care system, and this venue provides a way of “giving back” to society. This research also provides an opportunity for us to share experiences and help improve the health care system for others. We hope to further advocate for the patients’ interest and ensure accountability, providing a practical perspective to the research study.

We have participated as the sole patient and caregiver representatives in quarterly meetings with the overall team of iCOACH investigators. There have also been two three-day, in-person study team meetings. From time to time we are called upon to share our thoughts with the team, usually on specific areas that involve the patient/caregiver experience. We have also provided direct feedback on patient and caregiver surveys and interview guides used by the team.

## How to Ensure a More Successful Engagement of Patients and Caregivers on a Research Team

The experience for all parties has been a continuous learning curve and is evolving. Although our experience has been mostly positive, there have been a number of challenges. This is an entirely new experience for us and there has been much to learn in order just to participate. It has also been a challenge for the professional researchers to engage lay people in their research. We offer here some suggestions for ways to improve the present platform to become more meaningful.

At the outset of the study, we felt that we could not fully participate in team meetings because we did not understand the jargon and acronyms peculiar to the research community. Further, we were unfamiliar with government policy and the component parts of the health care system. It was somewhat intimidating and made it difficult to know how to engage. Training courses for patients and caregivers are needed at the beginning of a research project such as this. At the very least, a list of resource materials should be offered. The patient and caregiver advisors and the other members of the research team need to be made aware of the purposes of the engagement and role of the patient/caregiver.

We also suggest a small group involving patients/caregivers that meets separately from the full team. The purpose of this group would include such items as explaining jargon and acronyms and explaining government policy and the health care system. We could discuss patient/caregiver perspectives and give and receive feedback, including what our ongoing role in the project should be. We could also obtain ongoing explanations and updates about the project.

## The Hansons’ Attributes for Success in a Health Care System: A Personal Perspective

The focus for the research team is to improve the implementation of integrated care for older adults, and we turn now to reflections on our experiences with the health system that highlight important opportunities to improve integration. Over the years, we have identified eight distinct attributes that create an exemplary health care plan for us. When working well, they contribute to a successful health care outcome. The first two have been the most important:

1. Caregiver: A caregiver is absolutely essential in a complex case. This is someone who can speak for you when you can’t and who genuinely cares. Where a caregiver is absent, the role of a case manager becomes even more essential.
2. Case Manager: This role provides a central focal point with which the patient can interact in the health care system, ensuring that connections to services are made and referrals to other medical professionals are expedited. Trust and confidence in this individual is essential. A person who is a medical doctor, while not essential, is preferable. Goal congruence is important.
3. Ease of Access: This includes public universality at a reasonable cost for medically necessary services as well as private medical plans for additional supportive care. In our case, accessibility is strengthened by our close physical proximity to one of the largest medical facilities in the country with most specialties under one roof. Access to multiple medical professionals is an important component of integrated care.
4. Patient Engagement: This involves patients taking charge of their own care and lifestyle. Outcomes are more successful if, for example, one takes an active interest by researching current care for one’s specific conditions and concerns. We have found that when you see a health care professional, it is best to come prepared with an agenda that may include a list of the problems to be solved and questions to be answered in the limited time available to you.
5. Good Communication: We also include information technology under this heading. It is not only important to us that all the health professionals have and share the same information, but also that electronic and other systems are able to interact with each other.
6. Lab Results and Reports that are Informative and Timely: We have found that by having access to our own medical records, we are better prepared to discuss issues with the doctor the same or next day.
7. Home Care Services: This has been one of our most successful examples of integrated care. On several occasions, we have experienced the successful bridge and transition between the hospital and home settings. This is a win-win as the patient is much happier being treated at home and it is very cost effective for the health care system. We also note that these services have supported the caregiver function as well.
8. Mental Health Needs are Recognized: One should feel that you are in control of your medical problems, rather than that your medical problems are in control of you.

## What Can Be Improved in the Health Care System?

While elements of a successful health care system that have worked for us are noted above, we have also experienced inadequate care and offer suggestions for improvement.

As medicine has become increasingly specialized and patients have multiple health care problems, it seems to us that fewer practitioners are looking after the “whole” patient. Hence the need and importance of the “case manager” function in complex situations. In this function, the case manager should have ready access to other health specialists and should coordinate prescriptions, tests, and expensive technologies.

We need improved integrated electronic medical records to communicate between doctors, hospitals, specialists, and community support that are also accessible to patients. This would help prevent costly duplications and would improve service.

As hospital care becomes ever more expensive and care is shifted to the community, more adequate home and community care is needed. This should also include increased resources and support for caregivers, who are often seniors themselves and susceptible to “burnout”. One of the most apparent gaps identified by the research team is the lack of supports for caregivers.

Patients, where possible, must be more involved in, and take greater responsibility for, their health needs. There are few resources or specific programs offered to patients with the explicit goal to raise their capabilities and skills for self-management. The patient (and caregiver) must be involved in the process. Providers need to have tools and capabilities to involve, educate, and support patients and caregivers and to be accountable for this activity.

## Looking Ahead

To the extent that integrated care is present in the Ontario health care system, it has served us well. However, improvements as noted can and likely will be made, driven in part by the necessity of cost considerations. Patients should be engaged in these improvements and in the development of research to implement such changes.
